# Timing of Cefazolin Prophylaxis in Clean Surgery: Prophylaxis Administered After Incision Results in a Higher Surgical Site Infection Rate

**DOI:** 10.7759/cureus.113056

**Published:** 2026-07-20

**Authors:** Arthur J Morris, Tanya M Jackways, Jane Barnett, Christopher M Frampton

**Affiliations:** 1 Infection Prevention, Southern Cross Healthcare, Auckland, NZL; 2 Infection Control, Southern Cross Healthcare, Auckland, NZL; 3 Department of Medicine, University of Otago, Christchurch, NZL

**Keywords:** cefazolin, healthcare-associated infections, quality improvement, surgical antimicrobial prophylaxis, surgical site infection, surveillance

## Abstract

Introduction: The timing of antibacterial prophylaxis in the hour before incision remains undecided because of inadequate clinical outcome data. The purpose of this study was to determine if the timing of cefazolin prophylaxis in the hour before surgery influences the surgical site infection (SSI) rate in clean procedures.

Methods: We conducted a prospective SSI surveillance in 16 Southern Cross Healthcare hospitals in New Zealand, from 2005 through 2023. The timing of cefazolin antibacterial prophylaxis was recorded, in minutes, with respect to incision time. Patients were followed for 30 days after surgery. Standard definitions for SSIs were used. The SSI rate with respect to the timing of antibacterial prophylaxis was analysed in 10-minute periods. Uni- and multivariable analyses were performed.

Results: A total of 52,368 procedures had the timing of prophylaxis recorded in minutes against incision time. There were 694 (1.3%) SSIs. For all procedures, the lowest SSI rate was for prophylaxis given 21-30 minutes before surgery; however, after multivariate analysis, timing within the hour before incision was not significant. Prophylaxis given after incision had a higher SSI rate, with an odds ratio of 1.8 (95% CI: 1.2-2.8, P = 0.01).

Conclusion: The SSI rates for prophylaxis increased when cefazolin prophylaxis was given after incision, but no optimal time before incision was observed. The results may not be applicable to non-clean procedures or non-cefazolin prophylaxis.

## Introduction

More than 300 million surgical procedures are performed annually, and even if the surgical site infection (SSI) rate were only 1%, this would result in more than 9,000 SSIs each day [1]. These infections cause significant morbidity and divert resources away from other care. A cornerstone component of perioperative care bundles to reduce SSIs is the timely administration of surgical antimicrobial prophylaxis (SAP) [2-9]. The principal aim is to achieve an adequate free antibacterial tissue concentration from the start of the procedure [2].

For most situations, contemporary SAP guidance is for prophylaxis to be given in the hour before incision, but considerable variation exists [4-9]. For primary joint arthroplasty and cardiac surgery, 30-60 minutes before incision is recommended [10,11].

The Southern Cross Healthcare surgical hospital network has had a prospective SSI surveillance programme since 2002. The programme has observed a reduction in the SSI rate associated with improved adherence to interventions aimed at reducing SSI, namely, timing and dose of SAP and use of alcohol-based skin preparations for the incision site [12]. Because of the unsettled question on timing in the hour before incision, the data from this programme were analysed to see if there was evidence for an optimal time for cefazolin SAP in the hour before incision in a range of commonly performed clean procedures.

## Materials and methods

Surveillance

The SSI surveillance programme was developed from 2002 through 2003 at three hospitals and involved a small number of procedures. In 2004, after education and training, all 10 network hospitals started surveillance for SSIs. In addition, six joint venture hospitals joined the surveillance programme during the study period. In terms of data collection, procedures have a standard information dataset recorded, including patient sex, weight, American Society of Anesthesiologists (ASA) physical status score, duration of procedure, choice, timing and dose of SAP used, use of alcohol-based skin preparation, body mass index (BMI in kg/m^2^), presence of SSI, and type of SSI (superficial, deep incisional, or organ space). All details were entered into our bespoke database. Antibiotic prophylaxis was administered by a short intravenous push, not by infusion. The time for prophylaxis and incision was recorded using the 24-hour clock, allowing the time in minutes between the events to be calculated. Data were collected prospectively on the procedures chosen to be included by the network hospital. Procedures reflected the hospital’s case mix. Acute or trauma cases are not treated in our hospitals.

The programme aims to reduce patient harm by promoting compliance with measures known to reduce SSIs, e.g., correct dose and timing of surgical prophylaxis and use of alcohol-based skin preparations [12]. Continuing education on National Healthcare Safety Network (NHSN) definitions and surveillance methods was provided to hospital infection prevention and control nurses at network infection control training workshops. Quality improvement training on using the PDSA (Plan, Do, Study, Act) improvement model to develop, test, and implement changes leading to improvement was also provided at the workshops. Local quality improvement activities, using the PDSA improvement model, included three key change projects designed to improve adherence with prophylactic antibiotic dose of 2 to 3g of cefazolin, rather than 1gm; administer prophylaxis “on time”, i.e., within 60 minutes of incision, and ideally not less than 10 minutes before incision; and promote the use of an alcohol-based skin preparation for procedures with skin incisions, i.e. alcohol with either chlorhexidine or povidone-iodine. All three interventions were promoted starting from 2010 [12].

SSI definitions and case ascertainment

The CDC's NHSN definitions for SSIs were used, except that the assessment for infection was made at 30 days after the procedure [13,14]. Superficial incisional SSIs involve only the skin and subcutaneous tissue of the incision and must occur within 30 days of the operative procedure. Deep incisional SSI must involve deep soft tissues of the incision (e.g., fascia and muscle layers) and deeper infection is still considered organ/space SSI. Patients were informed during their stay that they would be contacted by either mail or phone to seek information on their wound. Responses suggesting an SSI were followed up with the patient’s primary care provider and/or surgeon to obtain relevant details. An SSI was only recorded if the signs and symptoms met the NHSN definition, e.g., a stitch abscess (minimal inflammation and discharge confined to the points of suture penetration) did not meet the SSI definition. For cases in which there was doubt about the presence of an SSI, authors experienced in applying the definitions (AJM, TMJ, and JB) were consulted for advice. From 2010, we recorded whether the SSI had resulted in admission to hospital. If the patient's 30-day feedback was not obtained, the procedure was excluded from the analysis.

Statistical analysis

The study cohort consisted of clean procedures performed from 2005 through 2023 with primary skin closure, receipt of cefazolin prophylaxis at a known time relative to the incision, and completed 30-day wound follow-up information. Varicose vein operations were excluded because the number and type of incisions were not known. Non-clean and laparoscopic procedures were also excluded.

The univariable associations between demographic, clinical, and surgical features and cefazolin prophylaxis timing and SSI were summarised as odds ratios (ORs) with 95% confidence intervals. The associations identified from the univariable analyses, the available sample size, and the established relevance with SSI were all considered in identifying candidate risk factors to be entered into a multivariable logistic regression model to determine the independent associations with SSI. To accommodate the inter-relationships amongst the independent variables in these models, forward and backward regression approaches were utilised to derive a robust final multi-variable model. The time of prophylaxis was categorised into 10-minute periods, as these represent clinically relevant time intervals while retaining an adequate number of procedures for each period. Timing was also analysed as being on time (1-60 minutes before incision), early (given more than 60 minutes before incision) or late (given at or after incision). The time interval with the lowest SSI rate was used as the reference group to generate ORs compared to other time intervals. A two-tailed P-value < 0.05 was considered statistically significant. All analyses were undertaken using SPSS Statistics for Windows, version 28.0 (IBM Corp., Armonk, NY).

Ethics

Under New Zealand Health and Disability Ethics Committee guidelines, formal ethical committee review is not needed for this type of quality improvement-related audit.

## Results

**Figure 1:**
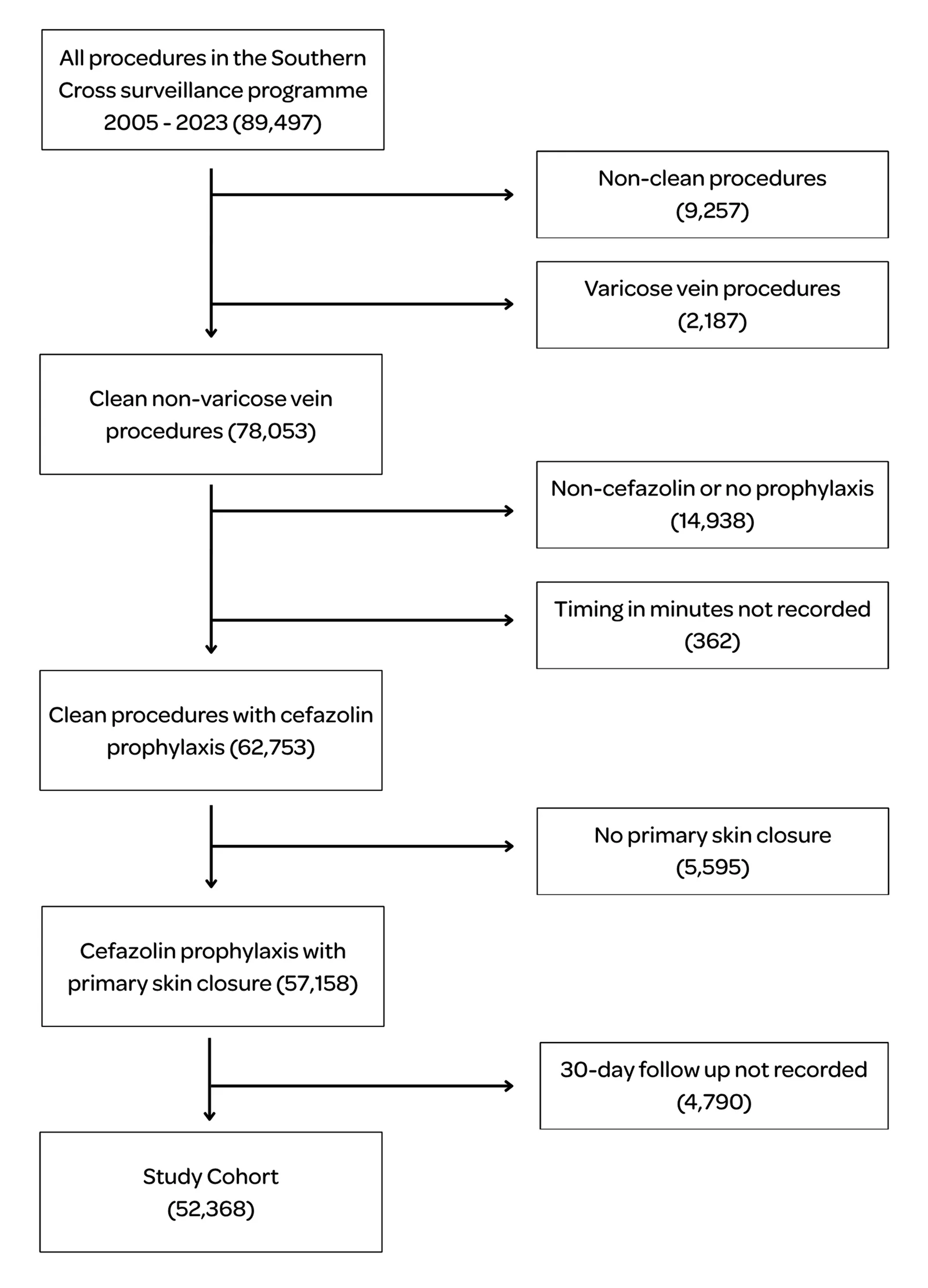
Filtering process used to derive the study cohort of clean procedures receiving cefazolin prophylaxis.

There were 89,497 operations included in the SSI surveillance programme from 2005 through 2025. The final cohort analysed included 52,368 procedures with primary skin closure and completed 30-day wound follow-up (Figure 1). Cefazolin was administered alone in 47,483 (91%) procedures, in combination with gentamicin in 4,630 (9%), and with another antibiotic in 255 (0.5%).

Procedure groups are shown in Table 1. Most cases were of orthopaedic procedures (84%), followed by breast (7%) or herniorrhaphy (6%) procedures. Breast surgery included mastectomy, reduction, and other procedures. Hernia repairs were mainly inguinal and umbilical or paraumbilical. Orthopaedic surgery was dominated by arthroplasties (87%) (Table 1). There were 694 SSIs, including 515 (74%) superficial, 106 (15%) deep, and 73 (11%) organ space. From 2010 (when monitoring of readmission to the hospital was in place), there were 467 SSIs, and at least 236 (51%) required readmission for management, including 93 (29%) of 319 superficial infections.

**Table 1:** Clean procedures receiving cefazolin prophylaxis. ^a^ Includes mastectomies (1,230, 35%), reductions (892, 26%), biopsies (509, 15%), augmentation (347, 10%), reconstruction (201, 6%), and others (284, 8%). ^b^ Includes laparotomies, biopsies, and cyst excisions. ^c^ Includes laparotomies and endometriosis procedures. ^d^ Includes cochlear implants, neck lymph node/mass excisions, and submandibular and parotid gland procedures. ^e^ Inguinal (1,750, 59%), umbilical/paraumbilical (801, 27%), incisional (159, 5%), and other or not stated (249, 8%). Includes mesh repairs. ^f^ Arthroplasties (37,910, 87%) (hip = 19,398, knee = 16,938, shoulder = 1,227, other joints = 347), knee ligament repairs (1,174, 3%), and other procedures (4,689, 11%) (e.g., carpal tunnel, removing metalware, fusions, laminectomies, discectomies, and arthrodeses). ^g^ Includes facelifts, liposuction, scar revisions, and skin grafts. SSI: surgical site infection.

Procedure groups	Total	SSI (N)	SSI (%)
Abdominoplasty	237	27	11.4
Breast^a^	3,477	108	3.1
Cardiac	293	26	8.9
General surgery^b^	186	1	0.5
Gynaecology^c^	182	4	2.2
Head and neck^d^	360	5	1.4
Herniorrhaphy^e^	2,959	32	1.1
Orthopaedic^f^	43,774	477	1.1
Plastic^g^	177	7	4.0
Thyroid/parathyroid	467	1	0.2
Vascular	226	6	2.7
Other	30	0	-
Total	52,368	694	1.3

Patient characteristics and univariable analysis of risk factors for SSI are summarised in Table 2. The highest risk factor for SSI was obesity. Late SAP had the highest SSI rate, with an OR of 2.4 (95% CI: 1.8-3.2, P < 0.001). The lowest SSI rate was observed when prophylaxis was administered 21-30 minutes before incision, with SSI rates increasing as SAP was administered closer to the time of incision (Table 2).

**Table 2:** Risk factors for surgical site infection for clean procedures receiving cefazolin prophylaxis. ^a^ The American Society of Anesthesiologists (ASA) physical status score. ^b^ On time: 1-60 minutes before incision; early: more than 60 minutes before incision; late: at or after incision. ^c^ Early: more than 60 minutes before incision; late: at or after incision. Cases with missing data were excluded from the analysis for each descriptor. The unadjusted odds ratios with 95% confidence intervals and p-values are derived from univariable logistic regressions. SSI: surgical site infection.

Descriptor		Total	SSI (N)	SSI (%)	Odds ratio	95% CI	P-value
Sex	Female	26,781	372	1.4	Reference		
	Male	25,554	322	1.3	0.91	0.78-1.05	0.19
	Total	52,335					
Age	<65 years	24,397	377	1.5	Reference		
	≥65 years	26,897	303	1.1	0.73	0.62-0.85	<0.001
	Total	51,294					
Hair removal	None	33,634	464	1.4	Reference		
	Clipping	13,861	187	1.3	0.98	0.82-1.16	0.79
	Shaving	2,413	24	1.0	0.71	0.47-1.07	0.11
	Total	49,932					
Body mass index (kg/m^2^)	<25	6,224	43	0.7	Reference		
	25-<30	10,657	106	1.0	1.44	1.01-2.06	0.043
	30-<35	7,061	96	1.4	1.98	1.38-2.84	<0.001
	35-<40	2,989	53	1.8	2.59	1.73-3.89	<0.001
	≥40	1,294	32	2.5	3.65	2.27-5.78	<0.001
	Total	27,895					
ASA score^a^	1	43,559	531	1.2	Reference		
	≥2	8,370	155	1.9	1.53	1.28-1.83	<0.001
	Total	51,929					
Skin preparation	Alcohol-based	46,704	578	1.2	Reference		
	Non-alcohol	5418	109	2.0	1.64	1.33-2.02	<0.001
	Total	52,122					
On time prophylaxis^b^	Late	3,498	83	2.4	1.92	1.53-2.43	<0.001
	On time	48,253	602	1.2	Reference		
	Early	617	9	1.5	1.17	0.60-2.27	0.64
	Total	52,368					
Time of cefazolin prophylaxis with respect to incision time^c^	Late	3,498	83	2.4	2.42	1.83-3.22	<0.001
	1-10 minutes	13,318	178	1.3	1.35	1.07-1.71	0.012
	11-20 minutes	16,036	204	1.3	1.26	1.02-1.65	0.032
	21-30 minutes	11,783	117	1.0	Reference		
	31-40 minutes	4,830	69	1.4	1.45	1.07-1.95	0.016
	41-50 minutes	1,621	30	1.9	1.88	1.23-2.82	0.002
	51-60 minutes	665	4	0.6	0.60	0.22-1.64	0.32
	Early	617	9	1.5	1.48	0.75-2.92	0.26
	Total	52,368	694	1.3			

After multivariable analysis, late SAP remained a risk factor for SSI, but timing within the hour before incision was not (Table 3). Other independent risk factors for SSI were age <65 years, BMI ≥25, and use of a non-alcohol-containing skin preparation. The lower SSI rate for older age is explained by a much higher proportion of those aged ≥65 years having orthopaedic surgery, which had a low SSI rate (56% vs. 28%, respectively; P < 0.001).

**Table 3:** Independent risk factors and cefazolin prophylaxis after multivariate analysis. ^a^ Alcohol skin preparation (containing either chlorhexidine or iodine) vs. an aqueous skin preparation for the surgical incision site. ^b^ Early: more than 60 minutes before incision; late: at or after incision. ^c^ Too few surgical site infections (n = 4) for multivariate analysis. The adjusted odds ratios with 95% confidence intervals and p-values are derived from multivariable logistic regression. C-statistic (0.67). Events and sample sizes are reported in Table 2.

Descriptor		Odds ratio	95% CI	P-value
Age ≥65 years		0.77	0.61-0.99	0.039
Body mass index (kg/m^2^)	<25	Reference		
	25-<30	1.64	1.14-2.39	0.008
	30-<35	2.49	1.71-3.62	<0.001
	35-<40	3.21	2.11-4.89	<0.001
	≥40	3.98	2.43-6.52	<0.001
Alcohol skin preparation^a^		0.60	0.41-0.89	0.012
Time of cefazolin prophylaxis with respect to incision time^b^	Late	1.81	1.15-2.84	0.01
	1-10 minutes	1.12	0.78-1.61	0.53
	11-20 minutes	1.25	0.88-1.76	0.21
	21-30 minutes	Reference		
	31-40 minutes	1.40	0.87-2.27	0.16
	41-50 minutes	1.63	0.83-3.29	0.15
	51-60 minutes	-^c^		
	Early	0.88	0.35-2.22	0.79

## Discussion

In this prospective cohort, no ideal time for administering cefazolin SAP was identified in the hour before surgery. Late SAP was a risk factor for a higher SSI rate (OR = 1.8).

It is difficult to compare these results with earlier publications. There are significant differences in design, size (most had fewer than 6000 procedures) [15-19], case mix (some only had one type of procedure [15,19,20], others had three to four groups [17,18,21-23]), proportion of non-clean procedures (0-24%) [15-17,23], inclusion of trauma or emergency operations, length of follow-up (30 days to one year), prophylactic agent(s) used, variables analysed, and analytical approaches taken. These results differ from those of the early report of Weber et al. [18], which observed higher SSIs when prophylaxis was administered within 30 minutes of incision, and the findings of Koch et al. [20], who observed a U-shaped curve for SSI rate and SAP timing in cardiac surgery. In the only randomised controlled trial addressing timing, Weber et al. [16] administered SAP in either the anaesthesia room (early, median administration time of 42 minutes before incision) or in the operating room (late, median administration time of 16 minutes before incision). They did not find any advantage in the early administration of cefuroxime prophylaxis. Recent reports on SSIs following cefuroxime prophylaxis broadly showed no differences in timing within the hour before incision (0-30 vs. 31-60 minutes) [21,24], although subgroup analysis in the large Swissnoso study observed an 11% lower SSI rate when prophylaxis was administered 10-25 minutes before incision versus 30-55 minutes before [24]. In a large Veterans Affairs cohort of 32,459 procedures, prophylaxis timing ranked 15th of the 16 variables and was not statistically significant in any model used [22]. In this cohort, no significant increase in SSI rates was observed as prophylaxis was administered closer to the time of incision.

Our results align with the only other study specifically looking at cefazolin SAP in clean procedures. That large prospective study reported on SSI outcomes following cefazolin SAP in 66,292 publicly funded hip and knee arthroplasties and cardiac surgery in New Zealand [25]. The cohort was followed for 90 days, and SSIs managed in the community were not included. After multivariable analysis, timing within the hour of incision was only significant if cefazolin was administered more than 51 minutes before incision [25]. While reporting on the same topic, i.e., cefazolin SAP in clean procedures, this study has additional features compared with the previous report and the literature. Other papers containing orthopaedic procedures focus primarily on hip and/or knee arthroplasties. Our cohort included other joint replacements (e.g., shoulder), as well as many orthopaedic procedures not included in other reports, such as spinal operations and soft tissue procedures (e.g., carpal tunnel), making it a broader sample of orthopaedic surgery. In addition, the cohort contains many surgical subtypes not often included in reports on clean procedures, e.g., breast, plastic, neurosurgery, vascular, and thyroid operations. Their inclusion makes our cohort a more representative sample of clean procedures getting antibiotic prophylaxis.

After multivariable analysis, there appears to be no difference in SSI outcome for cefazolin prophylaxis administered within 60 minutes of incision. This aligns with the WHO recommendation for prophylaxis with agents with a short half-life, such as cefazolin, to be administered within 60 minutes of incision [26].

This study has several strengths. Standard definitions for SSIs were used. It is one of the largest cohorts analysed for SSI outcome with respect to SAP timing, and there were almost 700 SSI events to analyse [15-23]. The size of the cohort allows insights not possible in smaller studies, i.e., the number of procedures analysed in each 10-minute period from 1 to 40 minutes before incision is greater than the total number of procedures in many previous reports [15,16,19,23]. Although dominated by orthopaedics, the cohort includes clean procedures not included in other studies, e.g., breast and hernia repairs. This and the inclusion of procedures from 16 hospitals suggest that the findings are likely to be generalisable.

This study also has limitations. SSIs occurring after 30 days were not included in this surveillance. All procedures were of a clean wound class, and these observations may not apply to non-clean surgery. Cefazolin was used alone in most procedures, and the potential impact of concomitant antibiotics was not assessed. As in almost all other reports, this study did not include dose as a variable for analysis and underdosing of cefazolin is associated with higher SSI rates [27,28]. Redosing during the procedure was not analysed. Finally, as an observational study, unmeasured residual confounding cannot be ruled out.

Although this was not a randomised trial, the findings suggest that there is little reason to refine the timing of prophylaxis within the hour before surgery. Nevertheless, administration within minutes of incision is unlikely to provide free tissue cefazolin concentrations effective against the expected pathogens in clean surgery.

## Conclusions

Interpretation of the literature on the timing of SAP is difficult because of significant case-mix variation between studies, e.g., studies containing both clean and clean-contaminated procedures, as well as different antibiotics used. In this large prospective cohort, representing a broad range of clean procedures, there was no “better time” to administer cefazolin prophylaxis in the 60 minutes before incision in clean surgery. Late SAP was a risk factor for a higher SSI rate, with an OR of 1.8.

We therefore concur with others that there is no need to refine the administration of prophylaxis in the hour before incision. However, efforts should be made to ensure cefazolin prophylaxis is administered before incision. Our findings may not apply to non-clean surgery or agents other than cefazolin.
